# Isolation and Characterization of an Agaro-Oligosaccharide (AO)-Hydrolyzing Bacterium from the Gut Microflora of Chinese Individuals

**DOI:** 10.1371/journal.pone.0091106

**Published:** 2014-03-12

**Authors:** Miaomiao Li, Guangsheng Li, Liying Zhu, Yeshi Yin, Xiaoliang Zhao, Charlie Xiang, Guangli Yu, Xin Wang

**Affiliations:** 1 Shandong Provincial Key Laboratory of Glycoscience and Glycotechnology, and Key Laboratory of Marine Drugs of Ministry of Education, Ocean University of China, Qingdao, Shandong, China; 2 State Key Laboratory of Breeding Base for Zhejiang Sustainable Pest and Disease Control, Institute of Plant Protection and Microbiology, Zhejiang Academy of Agricultural Sciences, Hangzhou, Zhejiang, China; 3 Key laboratory for Food Microbial Technology of Zhejiang Province, Zhejiang Academy of Agricultural Sciences, Hangzhou, Zhejiang, China; 4 State Key Laboratory for Diagnosis and Treatment of Infectious Diseases, First Affiliated Hospital, Zhejiang University, Hangzhou, Zhejiang, China; CNR, Italy

## Abstract

Agarose (AP) from red algae has a long history as food ingredients in East Asia. Agaro-oligosaccharides (AO) derived from AP have shown potential prebiotic effects. However, the human gut microbes responsible for the degradation of AO and AP have not yet been fully investigated. Here, we reported that AO and AP can be degraded and utilized at various rates by fecal microbiota obtained from different individuals. *Bacteroides uniformis* L8 isolated from human feces showed a pronounced ability to degrade AO and generate D-galactose as its final end product. PCR-DGGE analysis showed *B. uniformis* to be common in the fecal samples, but only *B. uniformis* L8 had the ability to degrade AO. A synergistic strain, here classified as *Escherichia coli* B2, was also identified because it could utilize the D-galactose as the growth substrate. The cross-feeding interaction between *B. uniformis* L8 and *E. coli* B2 led to exhaustion of the AO supply. *Bifidobacterium infantis* and *Bifidobacterium adolescentis* can utilize one of the intermediates of AO hydrolysis, agarotriose. Growth curves indicated that AO was the substrate that most favorably sustained the growth of *B. uniformis* L8. In contrast, κ-carrageenan oligosaccharides (KCO), guluronic acid oligosaccharides (GO), and mannuronic acid oligosaccharides (MO) were found to be unusable to *B. uniformis* L8. Current results indicate that *B. uniformis* L8 is a special degrader of AO in the gut microbiota. Because *B. uniformis* can mitigate high-fat-diet-induced metabolic disorders, further study is required to determine the potential applications of AO.

## Introduction

Agar, which is extracted from marine red seaweeds such as *Gelidium* and *Gracilaria*, is a linear polysaccharide made up of 3, 6-anhydro-L-galactose (A) and D-galactose (G) units alternately linked by α-(1, 3) and β-(1, 4) glycosidic bonds. Marine red algae containing agarose (AP) have a long history of use as food ingredients in East Asia. Agarose has also been widely used in the food industry as a thickener, stabilizer, and gelling agent. Worldwide production of agarose is estimated at about 20,000 tons annually, most of which goes to the food industry. So far, agarose has been used in large variety of foods, including fruit drinks, sweets, dessert, jelly, ice cream, and ham. Because dietary ingredients can affect the diversity and metabolic function of the human intestinal microbiota [1], it is desirable to understand whether agarose can be degraded or utilized by human gut microbiota.

Although agarose is a common additive in the modern food industry with a long history of use, little information is available regarding the bacterial strains of human gut microbes that can degrade the substrate. So far, the majority of agar-hydrolyzing bacteria are *Agarivorans*, *Alteromonas*, *Pseudoalteromonas*, *Saccharophagus*, or *Vibrio*, which are mainly isolated from marine environments [2]–[6] but can also be found elsewhere. Hehemann *et al.* screened the growth capacity of gut bacteria on red algal galactans and reported that *B. uniformis* NP1 could grow on agarose plates [7]. However, whether *B. uniformis* is the primary agarose degrader and whether any other colonic microbiota are involved in the degradation process have not been determined.

Recently, evidence has shown that agaro-oligosaccharides (AO) can act as a source of soluble fiber that may exert potential prebiotic effects [8]–[9]. Hu *et al.* found that the neoagaro-oligosaccharides could stimulate the growth of bifidobacteria and lactobacilli in mice [8]. Ramnani *et al.* reported that agaro-oligosaccharides with molecular weights of 64.64 KDa could induce significant increases in the population of bifidobacteria in an *in vitro* fermentation system inoculated with the human intestinal microbiota [9]. In addition, agaro-oligosaccharides have shown a range of health-promoting activities, such as anti-tumor [10], anti-inflammatory [11]–[13] and anti-oxidant properties [14]. The specific beneficial effects of AO are usually associated with specific degrees of polymerization (DP). For example, Hu *et al.* found agaro-oligosaccharides with higher DP to show better prebiotic activity than smaller DP [8]. However, once the gut microbes had broken the substrates down to smaller carbohydrates, activity levels decreased or even disappeared. In this way, the degradation of AO by human colonic microbiota must be taken into account, if the beneficial impacts are associated with the degree of polymerization.

In the current study, the fermentability of AP and AO by the gut microbes from Chinese individuals were compared in batch culture fermentation. A specific AO-hydrolyzing bacterium here identified as *B. uniformis* L8 was isolated. An accompanying bacterium, *Escherichia coli* B2, capable of utilizing the end products of AO degraded by *B. uniformis* L8 was also found. The hydrolytic characteristics of *B. uniformis* L8 with respect to other marine carbohydrates were further investigated.

## Materials and Methods

### Origin of samples

A total of six healthy human volunteers (living in Hangzhou, China), ranging from 22 to 35 years old, were recruited for the current study. The donors had not received antibiotics, pro- or prebiotic treatment for at least three months prior to sample collection. All volunteers provided informed, written consent, and the study was approved by the Ethics Committee of the Zhejiang Academy of Agricultural Sciences.

### Bacterial strains

*Bifidobacterium adolescentis* 1.2190, *Bifidobacterium infantis* 1.2202, *Bifidobacterium longum* 1.2186, *Bifidobacterium bifidum* 1.2212, and *B. uniformis* 1.5133 were purchased from China General Microbiological Culture Collection Center.

### Preparation of marine poly- and oligosaccharides

Agarose was obtained from Qingdao Judayang Seaweed Co. Ltd., China. AO was prepared using a modified version of a previously described procedure [15]. Briefly, agarose was treated with 0.1 M HCL at 60°C for 1 h, and then the hydrolytic product was neutralized and passed through nanofiltration membrane (molecular weight cutoff was 200) to remove the salt. The final substrate was obtained by concentration with rotary evaporation and lyophilization. Molecular mass (M_W_) of AO was measured using high-performance liquid chromatography (HPLC) (Agilent 1260, U.S.) with a TSK 3000 column, detected using a RI detector and multiangle laser light scattering [16].

Other marine oligosaccharides used in the current study including κ-carrageenan oligosaccharides (KCO) which were kindly provided by Glycoscience and Glycoengineering Laboratory, Ocean university of China. Guluronic acid oligosaccharides (GO) and mannuronic acid oligosaccharides (MO) were obtained from Lantai Pharmaceutical Company (Qingdao, China).

### Batch culture fermentation of AO and AP with human fecal slurries

Batch culture fermentations were conducted using the procedure described by Lei *et al.* [17]. Briefly, the basic growth medium VI contained the following (g liter^−1^): yeast extract, 4.5; tryptone, 3.0; peptone, 3.0; bile salts No. 3, 0.4; L-cysteine hydrochloride, 0.8; NaCl, 4.5; KCl, 2.5; MgCl_2_·6H_2_O, 0.45; CaCl_2_·6H_2_O, 0.2; KH_2_PO_4_, 0.4; Tween 80, 1 ml; and 2 ml of a solution of trace elements [18]. To assess the degradation and utilization of AP and AO by human fecal microbiota, 5.0 g of AO or 1.0 g of AP were added as the sole carbon source. The media were adjusted to pH 6.5 before autoclaving. Fresh fecal samples were homogenized in stomacher bags with 0.1 M anaerobic phosphate-buffered saline (pH 7.0) to make 20% (wt/vol) slurries. Large food residues were removed by passing the mixture through a 0.4 mm sieve. The human fecal slurry (7 ml) was inoculated into the serum bottle containing 63 ml of growth medium and the bottle incubated at 37°C for 48 h in anaerobic chamber (anaerobic workstation AW 500, Electrotek Ltd., U.K.). One milliliter sample were removed at 0, 24, and 48 h for analysis of carbohydrate degradation. The pH value after 48 h fermentation was measured by pH probe (Eutech, Singapore).

### Thin-layer chromatography (TLC)

The degradation of marine poly- and oligosaccharides was confirmed by TLC analysis. Briefly, samples (0.2 µl) were loaded on a pre-coated silica gel-60 TLC aluminum plates (Merck, Germany). After development with a solvent system consisting of formic acid/n-butanol/water (6∶4∶1, v∶v∶v), the plate was soaked in orcinol reagent and visualized by heating at 120°C for 3 min.

### Total carbohydrate analysis

The total carbohydrate concentration in the fermentation samples was determined using the phenol-sulfuric acid method, as described previously, using D-galactose as standard [19]. Results are expressed as the mean amount of total carbohydrate remaining relative to the amount detected at 0 h.

### DNA extraction and quantitative PCR

Bacterial genomic DNA from fermentation samples obtained at 0 and 48 h was extracted using a QIAamp DNA Stool Mini Kit according to the manufacturer's instructions (Qiagen, Germany). The concentration of extracted DNA was determined using a NanoDrop ND-2000 (NanoDrop Technologies, U.S.), and its integrity and size were confirmed by agar gel electrophoresis (1.0%).

The major bacterial groups in the batch fermentation samples were assessed using quantitative PCR (qPCR) using a CFX96™ Real-time PCR Detection System (Bio-Rad, U.S.). Six pairs of primers were used for total bacteria, *Bacteroides-Prevotella* group, *Bifidobacterium*, *Clostridium* clusters XIVab, *Enterobacteriaceae*, and *Lactobacillus*. The primer sequences are given in Table S1. Each reaction was performed in duplicate in a volume of 20 µl in 96-well optical-grade PCR plates. The reaction mixture comprised 50 pmol of each primer, 10 µl SYBR green Real-time PCR Master Mix (Toyobo, Japan), and 20 ng DNA template. Amplifications were performed with the following temperature profiles: 1 cycle of 95°C for 3 min, 40 cycles of 95°C for 15 s, primer-specific annealing temperature for 35 s, and then 72°C for 30 s. To determine the specificity of the PCRs, a melt-curve analysis was carried out after amplification by heating the PCR mixtures slowly from 60°C to 95°C. Fluorescence was assessed at 0.5°C intervals, 10 s per decrement. A quantitative analysis of unknowns was made using standard curves, which were made from known concentrations of plasmid DNA containing the corresponding amplicon for each set of primers [20].

### Isolation and identification of an AO-degrading bacterium

Two grams of fresh fecal samples No. 2 and No. 5 were homogenized separately in stomacher bags with 10 ml of 0.1 M anaerobic phosphate-buffered saline (pH 7.0) to produce 20% (wt/vol) slurries. Then 7 ml of each slurry was inoculated into 63 ml of VI growth medium containing 5 g/L of AO. After incubation in medium at 37°C for 24 h in an anaerobic chamber, fermentation samples were spread on an AO agar plate (basic growth medium VI plus 5 g AO and 15 g agar) using a 10-fold dilution method. Total 34 (No. 2) and 37 (No. 5) colonies were obtained from 7-fold dilution plates. Among them, 30 single colonies were randomly picked and re-inoculated into AO growth medium. AO degradation was assessed using TLC analysis of the supernatant. Positive colonies were further purified by repeating the 10-fold dilution method.

The isolates that demonstrated an ability to degrade AO were identified by sequencing their 16S rRNA gene. In brief, genomic DNA was extracted and the 16S rRNA gene was amplified by PCR with primers 27F (5′-CAGAGTTTGATCCTGGCT-3′) and 1492R (5′-AGGAGGTGATCCAGCCGCA-3′) [21]. DNA sequencing was conducted by Shanghai Sangon Biotech Co., Ltd. (Shanghai, China). Bacterial species were identified using aligned 16S rRNA sequences with the BLAST server (http://blast.ncbi.nlm.nih.gov/Blast.cgi) of the National Center for Biotechnology Information (NCBI). Isolates that could not be clearly classified by their 16S rRNA were evaluated with VITEK 2 gram-negative bacterial identification cards (GN cards, Biomerieux, France) using a VITEK Compact automatic bacteria identification instrument (Biomerieux).

### PCR-DGGE analysis

For analysis of the microbial communities, the V3 region of the 16S rRNA gene (positions 341 to 534 of the *Escherichia coli* gene) of all fermentations at 0 and 48 h was analyzed using PCR-denaturing gradient gel electrophoresis (DGGE), as described previously [17], [22]–[23]. Genomic DNA from *B. uniformis* L8 served as the marker. DGGE was performed using a DCode universal mutation detection system (Bio-Rad, Hercules, CA, U.S.) in an 8% (wt/vol) polyacrylamide gel containing a linear 30%-to-60% denaturant gradient with a constant voltage of 200 V at 60°C for 4 h [23]. The gels were then visualized by staining with SYBR green I nucleic acid (Sigma, St. Louis, MO, U.S.) for 45 min and washed twice with deionized water. The similarities of the DGGE profiles were analyzed by using Quantity One software (version 4.6.1; Bio-Rad, Hercules, CA) with a match tolerance of 4%.

Blocks of polyacrylamide gels containing selected DGGE bands were cut with a sterile scalpel. The blocks were then transferred in 40 µl of sterile water, and the DNA of the bands was allowed to diffuse overnight at 4°C. The water containing the eluted DNA was used for the reamplification. The PCR products were purified and cloned in PMD-18T vector (Takara, China). Three colonies were selected per DGGE band and sequenced by Shanghai Sangon Biotech Co., Ltd. (Shanghai, China). Searches in the NCBI database were performed with the BLAST program to identify the closest known relatives of the partial 16S rRNA sequence obtained [24]–[25].

### Degradation of AO by bacterial type strains

*B. adolescentis* 1.2190, *B. infantis* 1.2202, *B. longum* 1.2186, *B. bifidum* 1.2212, and *B. uniformis* 1.5133 were incubated into the VI growth media containing 5 g/L of AO and incubated at 37°C in anaerobic chamber for 192 h. Samples were collected at 192 h and analyzed with TLC.

### Chemical structure analysis of AO derivatives

The intermediates generated from the AO degradation after 48 h of incubation with human fecal isolate was determined by gel filtration chromatography and analyzed using negative-ion electrospray ionization mass spectrometry (ESI-MS). In brief, after removing the bacteria by centrifugation, the supernatant was separated on a Superdex Peptide column [16]. Three fractions (A1, A2, A3) were obtained and the sequence of each fraction was determined on a Thermo LTQ Orbitrap XL instrument (Thermo Finnigan Corp, U.S.) [15], [26].

Because TLC analysis showed only one spot after 96 h of incubation with human fecal isolates, monosaccharides analysis was performed using pre-column derivation with 1-phenyl-3-methyl-5-pyrazolone (PMP) [27]. Briefly, the end products of AO were derivatized with PMP and then analyzed using HPLC on a XDB-C18 column with acetonitrile/phosphate buffer solution (18∶82, pH 6.7) at a flow rate of 1.0 ml/min at 30°C. The detection wavelength was set to 254 nm. The composition of the end products was determined by retention time, in comparison with monosaccharide standards (mannose, rhamnose, xylose, galactose, glucose, glucuronic acid; Sigma Company, China).

### Utilization of marine oligosaccharides and polysaccharides AO, AP, GO, MO, and KCO by human fecal isolates

The utilization of marine poly- and oligosaccharides, including AO, AP, KCO, GO, and MO, by human fecal isolates were conducted by measuring the bacterial growth rates and degradation rates in media containing the corresponding carbohydrates. The basic growth medium VI, as described above, contained 5 g each of KCO, GO, MO, and AO. Due to the gelling property of AP, only 1 g of AP was used to make growth media, so no growth curve experiments were performed. Human fecal isolates, which showed the ability to degrade AO, were inoculated into the growth media and incubated at 37°C in an anaerobic chamber for 192 h. Samples were removed at 0, 24, 48, 96, 144 and 192 h (GO, MO) and 0, 48, 96, 144 and 192 h (AO, AP, KCO) for analysis of carbohydrate degradation. In the growth media containing AO, KCO, GO and MO, bacterial growth was measured using optical density (OD) at 600 nm.

## Results

### Comparative utilization of AO and AP by six human fecal samples

**Figure 1:**
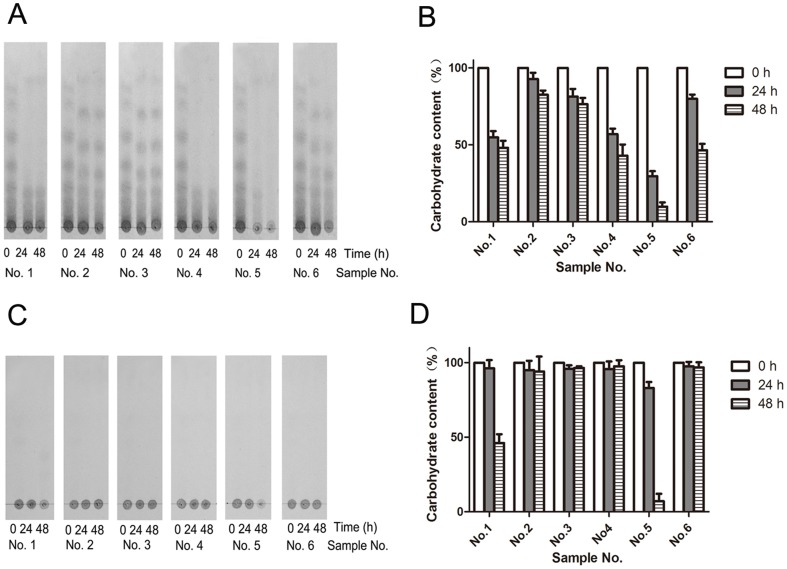
Degradation of agaro-oligosaccharides (AO) and agarose (AP) by six human fecal microbiota in the batch fermentation. A) TLC patterns of AO fermentation by six fecal slurries. The growth medium contained 5 g/L of AO and samples were collected at 0, 24, and 48 hours. B) The residual values of total carbohydrates in the batch AO fermentation. The results are expressed as the mean of the relative amount of original total carbohydrates from duplicate measures determined in three independent experiments. C) TLC patterns of AP fermentation. The growth medium contained 1 g/L of AP and samples were collected at 0, 24, and 48 hours. D) Residual values of total carbohydrates in the batch AP fermentation.

The AO used in the current study was a mixture of odd-numbered oligosaccharides, and the average M_W_ was approximately 5 kDa. In general, AO was degraded to smaller intermediates by all tested fecal samples. The molecular sizes of the intermediates generated by the fecal bacterial degradation were markedly different from the original substrates, as evidenced by TLC analysis (Figure 1A). In addition, the extent of AO degradation was found to vary significantly among the tested six human fecal samples. Fecal sample No. 5 was found to degrade and utilize all of the available AO; the substrate disappeared after 48 h of fermentation. Fecal samples Nos. 1, 4, and 6 had degraded some of the substrate after 48 h. Two fecal samples (Nos. 2 and 3) were only slightly able to degrade substrate (Figure 1A).

The AO degradation by six human fecal slurries was further confirmed by measurement of total carbohydrates concentrations after 24 and 48 h of fermentation. As shown in Figure 1B, only 30.7% and 12.5% of total carbohydrates remained in the growth media after 24 and 48 h of fermentation, respectively, with fecal sample No. 5. In contrast, the levels of total carbohydrates were 93.0% and 82.6% after 24 and 48 h of fermentation with fecal sample No. 2. Changes in pH were consistence with the degree of the AO degradation (Table S2). After 48 h of fermentation with fecal sample No. 5, the pH reached 5.39. Fecal samples Nos. 2 and 3 showed no significant changes in pH after 48 h of fermentation.

Degradation of AP by six human fecal samples is shown in Figure 1C and TLC analysis also showed various extents of degradation. Fecal samples No. 1 and No. 5 were able to degrade AP after 48 h of fermentation. Fecal sample No. 5 showed a faster rate of utilization than fecal No. 1. The other four fecal samples showed almost no degradation. The levels of total carbohydrates remaining in the growth medium after 24 and 48 h of fermentation are shown in Figure 1D. In consistent with the TLC results, the total carbohydrate concentrations in fecal samples No. 1 and No. 5 were 46.36% and 9.2%, respectively after 48 h of fermentation, but carbohydrates were showed hardly any degradation in the other tested samples.

### AO and the composition of human fecal microbiota community

To determine the effect of AO on the composition of fecal microbiota, changes in the populations of major bacteria from six fecal samples including *Bacteroides*, *Bifidobacterium*, *Clostridium* cluster XIVab, *Lactobacillus*, and *Enterobacter* were measured after 48 h of AO fermentation using qPCR with specific primers. As shown in Table S2, there was no obvious trend regarding groups of total bacteria or lactobacilli in any of the tested samples. Increasing trends were observed in all samples with respect to the populations of *Bifidobacterium* and *Clostridium* cluster XIVab. The numbers of *Bacteroides* showed a weak correlation with the degree of AO degradation. Fecal sample No. 5 showed the highest increase, although the change was statistically insignificant. A significant increase in the number of *Enterobacteriaceae* was also detected in fecal sample No. 5 after 48 h of AO fermentation. PCR-DGGE was also used to analyze effect of AO on the composition of fecal microbiota (Figure S1A). UPGMA analysis showed no obvious relatedness of PCR-DGGE profiling and the extents of AO degradation (Figure S1B).

### Isolation of the AO degradation bacteria

**Figure 2:**
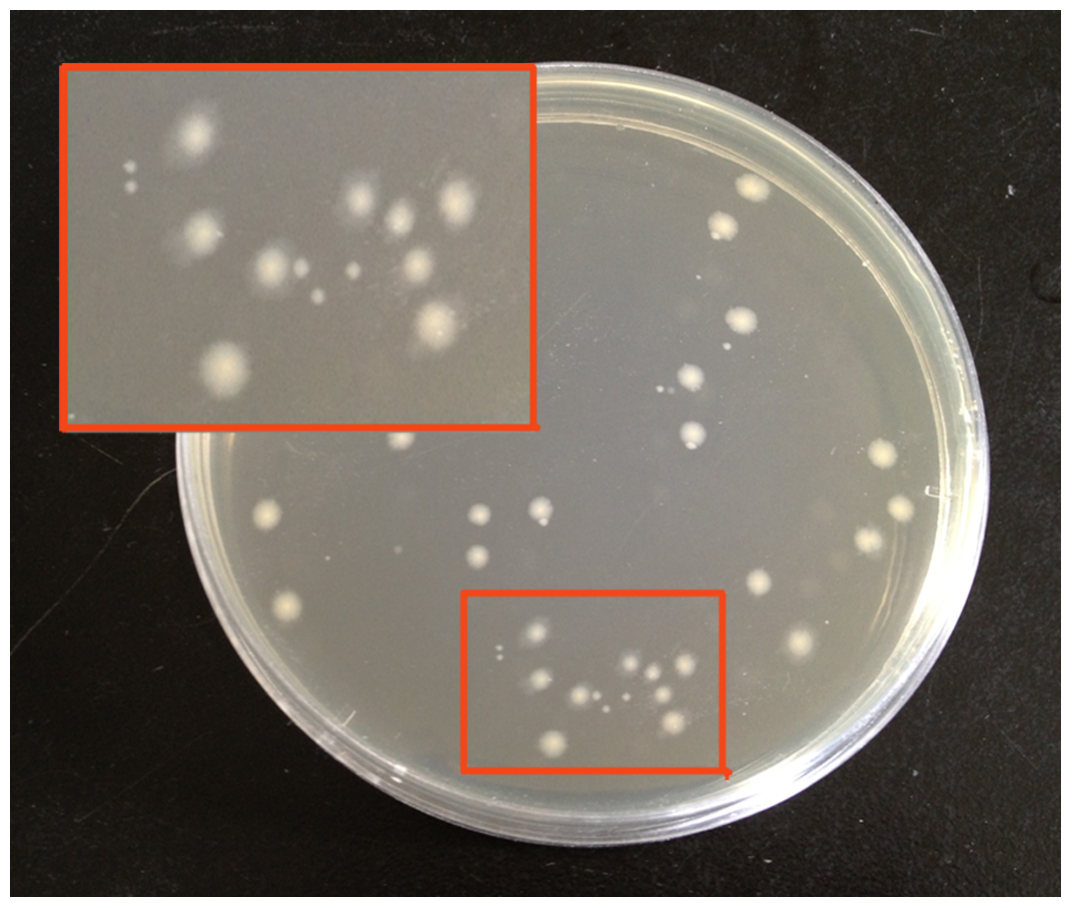
Colony morphology of the agaro-oligosaccharides (AO) degrading bacteria grown on an agarose plate. The small colonies were identified as *B. uniformis* L8 and the large colonies were identified as *E. coli* B2.

Because fecal sample No. 5 showed the highest rate of AO utilization, the fecal culture which had been grown in medium containing AO for 48 h was spread on AO agar plates for isolation of the AO hydrolyzing bacterium. A total of 34 colonies were detected on the 7-fold dilution plate, which was equivalent to 1.8×10^11^ CFU/g feces. Thirty colonies were picked up from the AO agar plates and re-inoculated into growth medium containing 5 g AO. TLC analysis was conducted after 72 hour incubation in AO containing medium. TLC patterns indicated that the AO substrate had completely disappeared from the supernatant of one isolate, suggesting that this colony could hydrolyze AO. The positive colony was then purified by repeated spreading on the AO agar plate in the anaerobic chamber. Two types of colonies were observed. One had a relatively slow growth rate and produced colonies with the smaller size, whiles another one showed fast growth (Figure 2). After sequencing their 16S rRNA, the small size colonies were identified as *B. uniformis* L8, showing 99% similarity under BLAST at the NCBI database, and the large colonies were classified as *Escherichia coli/Shigella dysenteriae*, showing 99% similarity. All sequences were submitted to NCBI under the accession number KF830695 for *B. uniformis* L8, and KF830694 for *E. coli* B2. The large colony was then further identified as *E. coli* B2 with 99% possibility using a VITEK Compact automatic bacteria identification instrument. The bio-reactions used for B2 identification are listed in Table S3.

**Figure 3:**
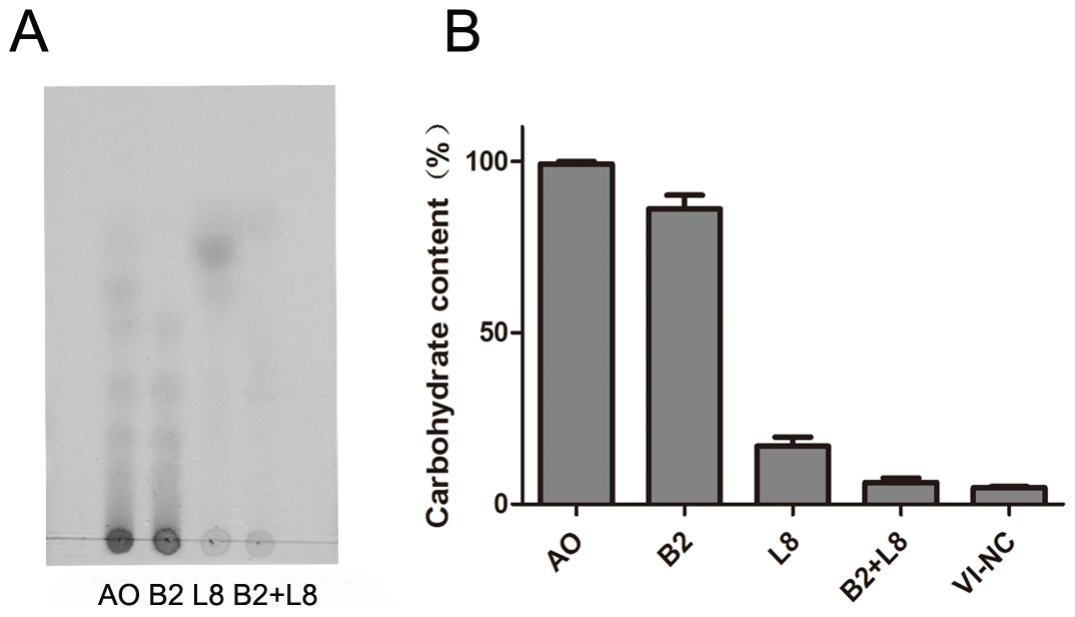
Degradation of agaro-oligosaccharides (AO) by *Escherichia coli* B2 and *Bacteroides uniformis* L8. A) TLC patterns after AO fermentation by *E. coli* B2 (B2), *B. uniformis* (L8) and B2 plus L8 grown together for 96 h. AO growth medium contained 5 g/L AO. Results shown are representative of three independent experiments. B) The residual values of total carbohydrates after AO fermentation by B2, L8, and B2 plus L8 for 96 h. The results are expressed as the mean of the relative amount of original total carbohydrate from duplicate measures determined in three independent experiments. VI-NC growth medium contained no carbohydrates.

The ability of *B. uniformis* L8 and *E. coli* B2 to degrade AO was tested by inoculating strains L8 and B2 into the AO growth media individually and together. TLC analysis indicated that *B. uniformis* L8 alone could degrade AO: the original substrates disappeared after 96 h of fermentation, showing a single spot (Figure 3A). In contrast, *E. coli* B2 could not degrade AO at all. When *B. uniformis* L8 and *E. coli* B2 were grown together in AO medium, the intermediates generated by L8 disappeared. Then the single spot generated by *B. uniformis* L8 was subjected to HPLC analysis. After comparison to monosaccharide standards, the end product of AO hydrolyzed by *B. uniformis* after 96 h was identified as D-galactose. Total carbohydrate concentrations in the media containing *B. uniformis* L8, *E. coli* B2, and both were also measured. In line with the TLC analysis, the remaining carbohydrate levels in *B. uniformis* L8, *E. coli* B2, and both were 17.2%, 86.0%, and 6.5% of the original concentration, respectively, suggesting that the presence of *E. coli* B2 allowed increased utilization of the end products generated by *B. uniformis* L8 (Figure 3B).

Similarly, sample No. 2 was also grown in medium containing AO for 24 h and spread on AO agar plates for isolation of the AO hydrolyzing bacterium. Thirty colonies were picked up from the AO agar plates and re-inoculated into growth medium containing 5 g AO. TLC analysis was conducted after 192 h of incubation in AO containing medium. One colony showed a weak ability to degrade AO (Figure S2A). It was classified as *Bacteroides dorei* by sequencing of its 16S rRNA, and the sequence was submitted to NCBI under accession number KJ145327.

### Distribution of *B. uniformis* in human fecal samples

Because the human fecal isolate, *B. uniformis* L8 demonstrated the ability to degrade AO and AP, the distribution of the strains was assessed across all testing samples. The PCR-DGGE profiles of fecal microbiota communities showed that a band produced by fecal samples No. 1, No. 2, No. 5, and No. 6 was located in a similar position to the band generated from the purified human isolate *B. uniformis* L8 (Figure S1A). After cloning and sequencing, the sequences of those bands were found to be identical to each other and to *B. uniformis* as identified by BLAST in the NCBI database. Although all bands except that produced by sample No. 5, whose intensity increased after AO fermentation, were identified as *B. uniformis*, the populations of *B. uniformis* among other tested samples were not significantly affected by the AO fermentation. For example, the band of *B. uniformis* disappeared in sample No. 1 after 48 h of fermentation with AO, but the intensity of the bands generated from samples No. 2 and No. 6 showed no change.

*B. uniformis* was found to be common in fecal samples No. 1, No. 2, No. 5, and No. 6 under PCR-DGGE analysis, but only fecal No. 5 was able to completely degrade AO, it is here proposed that the degradation of AO by *B. uniformis* L8 is strain-specific. To test this hypothesis, *B. uniformis* 1.5133 was obtained from national culture collection and grown in the VI growth media containing 5 g/L of AO for 192 h. The degradation of AO by *B. uniformis* 1.5133 was detected by TLC analysis. As shown in Figure S3, *B. uniformis* 1.5133 did not show any ability to degrade AO.

### Chemical structures of the intermediates produced by the degradation of AO by *B. uniformis*

TLC patterns showed the intermediates produced from the degradation of AO by *B. uniformis* to contain a range of fragments with different molecular weights after 48 h of incubation. However, the intermediates produced after 96 h of incubation consisted of only a single spot, which was identified as D-galactose by HPLC. The chemical structures of the intermediates at 48 h were further analyzed by ESI-MS analysis. Results showed that the composition of the intermediates from AO degradation by *B. uniformis* L8 after 48 h of fermentation mainly included D-galactose (DP = 1), agarotriose (DP = 3), and agaropentaose (DP = 5). Minor amounts of neoagarobiose (DP = 2) and agaroheptaose (DP = 7) were also observed (Table 1).

**Table 1:** Negative-ion LTQ-MS analysis of the fragments generated from agaro-oligosaccharide (AO) degradation by *B. uniformis* L8 at 48 hours.

Fraction	Found ions (Charge)	Theoretical H form	Assignment	
			DP	Sequences
A1	179.1(−1)	180.1	1	G
	323.1(−1)	324.1	2	A-G
A2	485.2 (−1)	486.2	3	G-A-G
A3	791.3 (−1)	792.3	5	G-A-G- A-G
A4	1097.4 (−1)	1098.3	7	G-A-G-A-G- A-G

G, β-1,3-D-galactose; A, α-1,4-L-3,6-anhydro-galactose.

### Degradation of AO by *Bifidobacteria*

**Figure 4:**
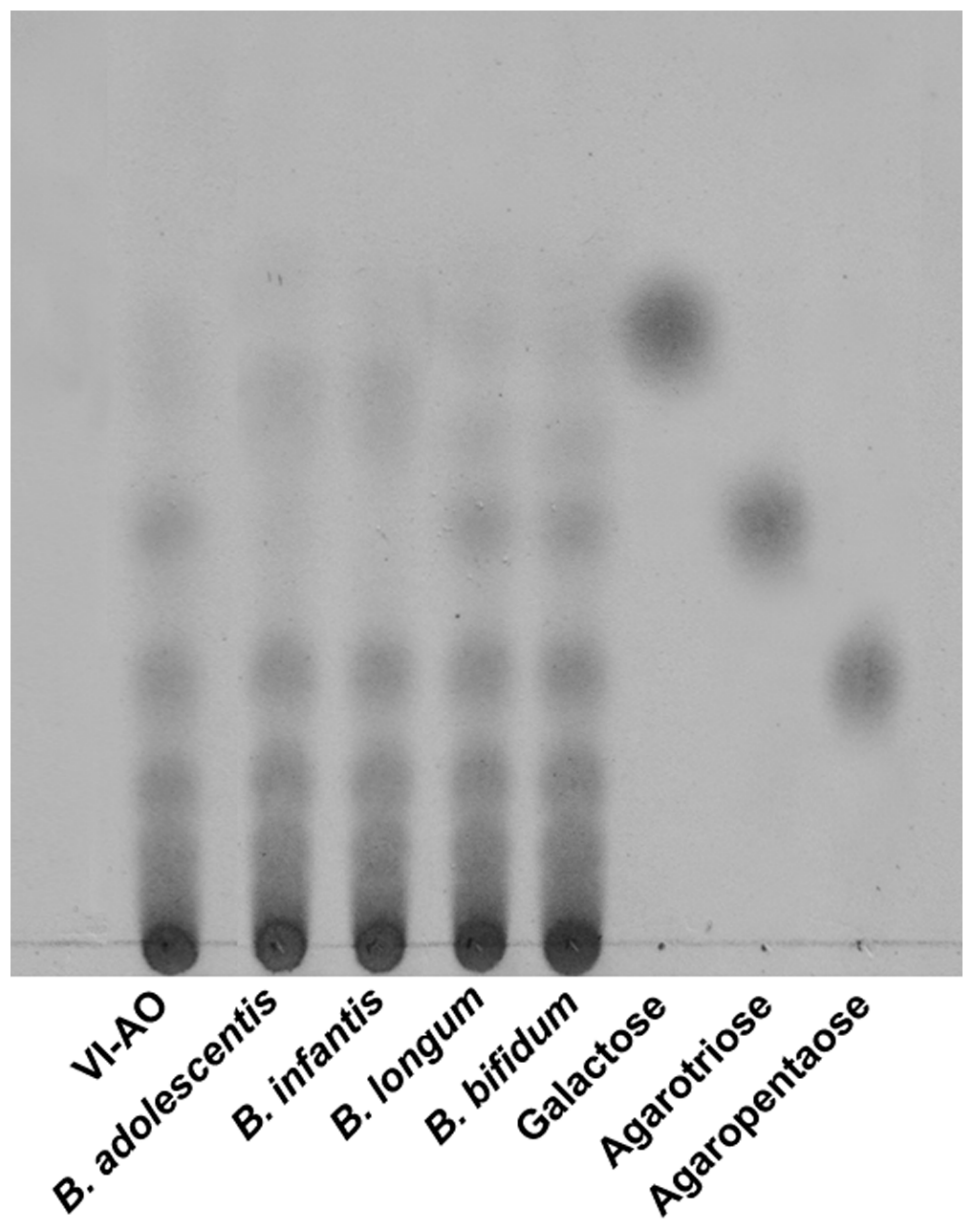
TLC analysis of degradation of agaro-oligosaccharides (AO) by *Bifidobacteria* after 192 h of culture.

Because the results of qPCR analysis showed that AO in the media could enhance the growth of bifidobacteria in the mixed fecal slurries, AO degradation and utilization by *Bifidobacteria* were tested. *Bifidobacteria* were grown in VI medium containing 5 g/L of AO for 192 h. Then TLC analysis was performed. Results showed none of *Bifidobacteria* strains to be able to degrade AO under these growth conditions. However, *B. adolescentis* and *B. infantis* were able to utilize agarotriose (DP = 3) which is a component of AO and the intermediates of AO hydrolysis. *Bifidobacterium longum*, and *Bifidobacterium bifidum* were not able to utilize any of the components of AO (Figure 4).

### Comparative utilization of other marine carbohydrates by *B. uniformis*

**Figure 5:**
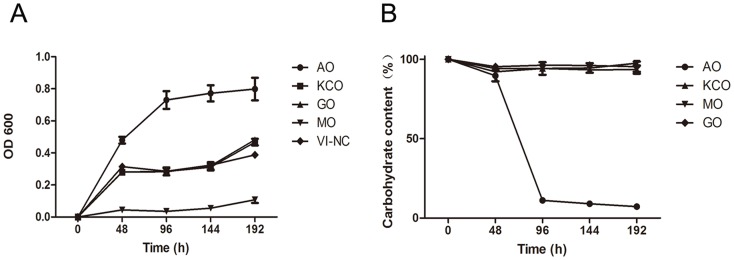
Growth curves of *B. uniformis* L8 in medium with different carbohydrates and total carbohydrates left in the medium during the growth of *B. uniformis* L8. A) Growth curves of *B. uniformis* L8 in medium containing 5 g/L of agaro-oligosaccharides (AO), κ-carrageenan oligosaccharides (KCO), guluronic acid oligosaccharides (GO), and mannuronic acid oligosaccharides (MO). VI-NC medium contained no carbohydrates. B) The relative amount of total carbohydrates left in the medium during the growth of *B. uniformis* L8. Results are the means of duplicate measures determined in three independent experiments.

The utilization rates of marine carbohydrates, including those of AO, AP, KCO, GO, and MO were measured by testing the growth curves of *B. uniformis* and its ability to degrade carbohydrates. Based on the results of TLC analysis (Figure S4), *B. uniformis* L8 was found to degrade AP into the fragments with small molecular weights after 96 h of fermentation, but did not break down KCO, GO, or MO even after 196 h of fermentation. Due to the gelling property of AP, the total carbohydrate level used in the AP media was only 1 g/L, unlike the 5 g/L used for KCO, GO, MO, and AO. In this way, the growth curves of *B. uniformis* L8 were only measured in the media containing KCO, GO, MO, and AO. The basic medium VI, which did not contain any carbohydrates (VI-NC), served as the control. As shown in Figure 5A, the most growth was detected in the medium containing AO, and the presence of KCO and GO was not found to affect bacterial growth. The presence of MO suppressed the growth of *B. uniformis* L8. Carbohydrate utilization by *B. uniformis* L8, as measured using the phenol-sulfate method, is shown in Figure 5B. As expected, the concentration of AO declined markedly after 96 hour incubation.

## Discussion

The human gut is inhabited by 10^14^ microbes, including of 1,000 to 1,150 phylotypes. Significant variations in the composition of colonic microbiota exist among individuals, and each person is estimated to have at least 160 bacterial phylotypes in the gut [28]–[31]. In this way, the ability to degrade and utilize complex polysaccharides may vary among the individuals due to the different compositions of gut microbiota. Six human fecal samples were examined, and the degradation of AO was found to vary. It was classified into four categories according to the degradation rates and fragmentation patterns. The organisms in the first category, as shown in fecal sample No. 5, can degrade and utilize AO and AP almost completely. In contrast, because fecal samples Nos. 2 and 3 were found to utilize the AO in less than 10%, they were placed in the second category. Although fecal samples Nos. 1, 4, and 6 were found to have degraded 50% of total AO after 48 h of fermentation, the different patterns of the residual fragments (Nos. 1 and 4 shared the same pattern, No. 6 showed another one) generated from AO degradation suggest that the bacterial communities involved in the degradation may differ. For this reason, they were believed to have at least two different utilization patterns. Higashimura *et al.* suggested AO may be a suitable supplemental therapeutic strategy for the treatment of inflammatory bowel diseases [11]. The present data indicate that the different degradation patterns produced by individual gut microbiota may affect the therapeutic impact. Certain groups of people can completely degrade AO to mono-sugar, but others cannot digest it at all. Based on the current discovery, the utilization rates and patterns of the complex polysaccharides and oligosaccharides by fecal microbiota may need to be determined prior to the use of any of these oligosaccharides as therapeutic agents for which the beneficial effect is associated with the degree of polymerization.

Bacteroides/Prevotella are the most numerous microorganism in the human gut [32] and they plays a key role in the degradation of seaweed polysaccharides. Recent metagenomic studies have reported that the β-agarase and porphyranase genes were present in *Bacteroides pleibeius* that were isolated from the gut microbiota of Japanese individuals [33]. In addition, *Bacteroides ovatus* was found to be able to degrade alginate, and *Bacteroides thetaiotaomicron* was found to utilize laminarin [34]. In the present study, the AO-degrading bacterium isolated from human fecal samples was also part of the genus *Bacteroides*. Based on 16S rRNA sequencing, this *Bacteroides* sp. from No. 5 was classified as *B. uniformis*, although *B. dorei* showed weak degradation.

*B. uniformis* is common in the human intestinal microbiota [35]–[36]. In the current study, *B. uniformis* was detected in 4 out of 6 fecal samples. However, only the *B. uniformis* isolated from fecal sample No. 5 showed itself to be involved in AO degradation. For example, the band of *B. uniformis* in fecal sample No. 1 disappeared after 48 h of AO fermentation and the intensity of bands from samples No. 2 and No. 6 did not increase. The AO degradation rates of samples No. 1, No. 2, and No. 6 reached 52.8%, 35.4%, and 52.3%, respectively. That indicates that other bacteria may also be able to utilize AO. The similar degradation patterns generated by *B. dorei* and by fecal No. 2 suggest that *B. dorei* may be key to AO utilization in fecal No. 2. The fact that *B. uniformis* 1.5133 did not degrade AO further confirmed that L8 is a special strain capable of degrading AO.

Ramnani *et al.* recently reported that agaro-oligosaccharides with a molecular weight of 64.64 KDa can enhance the growth of bifidobacterial *in vitro* [9]. The bifidobacteria population was found to increase in the current study. But bifidobacteria may not be the primary agarose degraders. The four bifidobacteria tested here were not able to hydrolyze AO at all. However, *B. adolescentis* and *B. infantis* were able to utilize agarotriose, which are the broken down fragments produced from the degradation of AO. It is therefore reasonable to propose that *Bacteroides* sp., but not bifidobacteria, plays the key role in the utilization of AO with higher DP. The broken-down fragments produced from the degradation of AO by *B. uniformis* can then be utilized by bifidobacteria in the gut, resulting in the increase in the bifidobacteria population.

Cross-feeding interaction is a common phenomenon between the members of colonic microbiota. In general, two distinct mechanisms of cross-feeding take place in the gut ecosystem, metabolic cross-feeding and substrate cross-feeding [37]. Metabolic cross-feeding involves the consumption of fermentation end products such as SCFA produced from one species, and substrate cross-feeding involves the consumption of a partially broken-down intermediate generated by one species during the degradation process of a complex carbohydrate. The degradation and utilization of AO is a synergistic process, involving different bacterial groups. *B. uniformis*, degrades of AO, breaking it down into small fragments with DP values ranging from 1 to 7. Bifidobacteria can utilize DP3 fragments, and *E. coli* can utilize DP1. Like bifidobacteria, *E. coli* cannot utilize AO by itself. When *E. coli* and *B. uniformis* are co-cultured, *E. coli* utilizes the D-galactose generated by *B. uniformis* as a source of carbon for its growth. Eventually, the cross-feeding phenomenon between *B. uniformis* and *E. coli* leads to the complete exhaustion of AO.

Unlike marine oligosaccharides such as KCO and GO, which do not influence the growth of *B. uniformis* L8, MO inhibits the growth of *B. uniformis* L8. In the present study, MO and GO are poly-β-D-mannuronic acid and poly-α-L-guluronic acid derived from alginate, respectively. Both have molecular weights of 2–3 KDa. The difference between them is that MO consists of 1→4 linked β-D-mannuronic acid, but GO is composed of α-L-guluronic acid, the C-5 epimer of β-D-mannuronic acid [38]. For this reason, the inhibition effect of MO on the growth of *B. uniformis* L8 is most likely due to its chemical structure. The mechanism of underlying anti-*B. uniformis* effect of MO needs further study.

*B. uniformis* CECT7771 demonstrated a stronger capacity to induce anti-inflammatory cytokine IL-10 production *in vitro* than other *Bacteroides* species. Cano *et al.* also reported that *B. uniformis* CECT7771 can significantly improve innate and adaptive defense mechanisms against infections in mice with high-fat-diet (HFD)–induced obesity when they were given oral *B. uniformis* CECT7771 for 7 weeks. These results suggest that *B. uniformis* CECT7771 may have beneficial roles in the defense against metabolic dysfunction caused by obesity [39]. In the current study, *B. uniformis* L8 was isolated from a fecal sample. This organism showed a pronounced ability to degrade AO and AP *in vitro*. A synergistic strain, *E. coli* B2, was also identified. HPLC analysis showed D-galactose to be the end product of AO degradation by *B. uniformis*. It can then be utilized by the chaperon strain *E. coli* B2. Because AO is the major carbohydrate selectively utilized by *B. uniformis* L8 and because *B. uniformis* can resolve obesity-related metabolic and immunological dysfunction in mice, AO may be suitable for therapy targeting obesity-related metabolic disorders. Further animal studies are required to evaluate the protective effects of AO and *B. uniformis* in organisms consuming a high-fat diet.

## Supporting Information

Figure S1 **PCR-DGGE Analysis of the microbial communities of six fecal slurries before and after AO fermentation.** A) PCR-DGGE files of six fecal slurries before and after AO fermentation. Samples were taken at 0 and 48 h. Bands a–h were cut out, colonized, and sequenced. B) The UPGMA analysis of PCR-DGGE profiles from six fecal samples before and after AO fermentation.
(TIF)

Figure S2 **TLC analysis of AO degradation by** ***B. dorei*** **after 192 h of fermentation.** A) TLC patterns after AO fermentation by *B. dorei*. B) The residual values of total carbohydrates after AO fermentation by *B. dorei*.
(TIF)

Figure S3 **TLC analysis of AO degradation by** ***B. uniformis*** **1.5133 after 192 h of fermentation.**
(TIF)

Figure S4 **Degradation of AO, AP, KCO, MO, and GO by** ***B. uniformis*** **L8 TLC patterns of AO, AP, KCO, MO and GO fermentation by** ***B. uniformis*** **L8.** Samples from AO, AP, and KCO fermentation were collected at 0, 48, 96, 144, and 192 h. MO and GO were collected at 0, 24, 48, 96, 144, and 192 h.
(TIF)

Table S1 **Primers used for qPCR.**
(DOCX)

Table S2 **Bacterial populations (log cells/ml) and pH values from six fecal slurries after batch fermentation of AO.** Samples were taken at 0 and 48 h of AO fermentation.
(DOCX)

Table S3 **Biochemical details of** ***E. coli*** **B2 determined with GN card by VITEK Compact automatic bacteria identification instrument.**
(DOCX)
