# Supplementary figures and images for: Isolation and Characterization of an Agaro-Oligosaccharide (AO)-Hydrolyzing Bacterium from the Gut Microflora of Chinese Individuals
Source: PLoS One. 2014 Mar 12;9(3):e91106. doi: 10.1371/journal.pone.0091106 (PMC3951304; doi:10.1371/journal.pone.0091106)

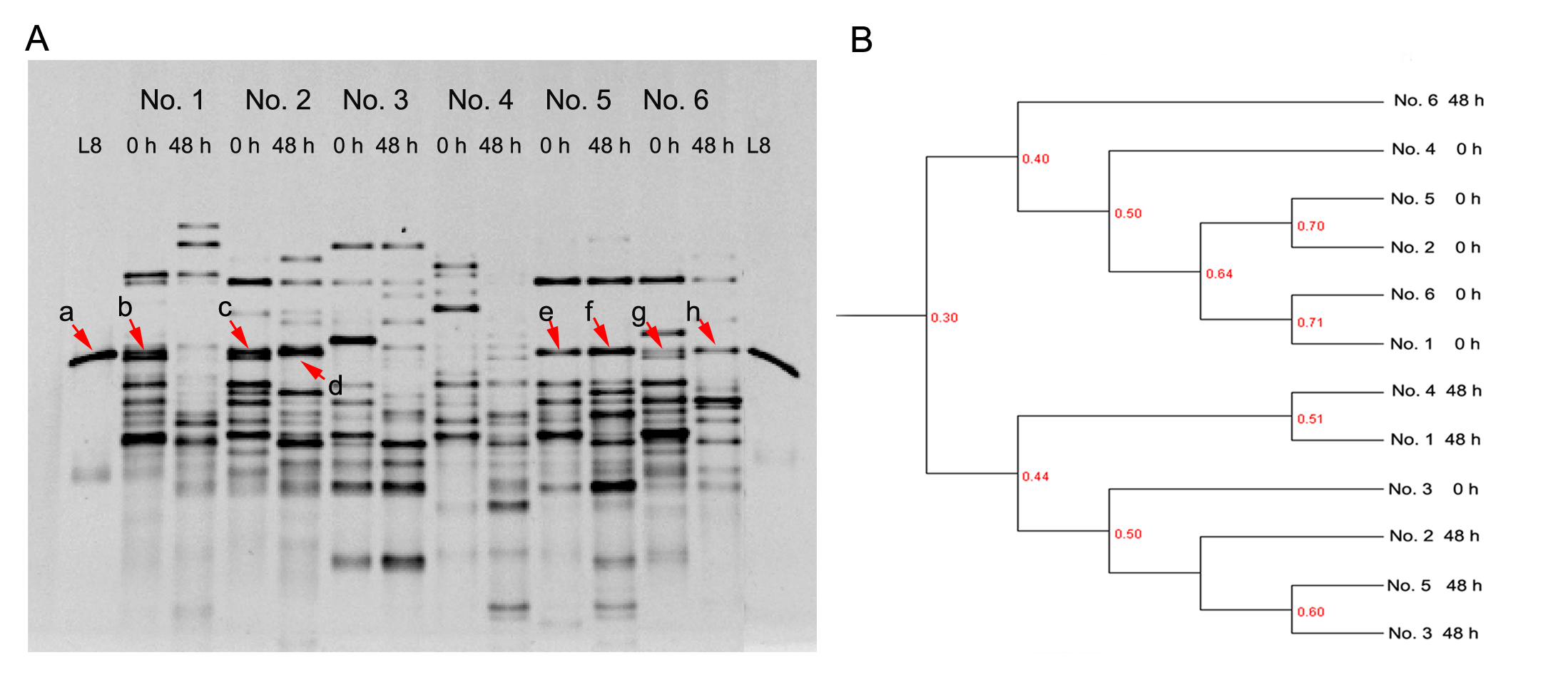

Supplement: Figure S1 — PCR-DGGE Analysis of the microbial communities of six fecal slurries before and after AO fermentation. A) PCR-DGGE files of six fecal slurries before and after AO fermentation. Samples were taken at 0 and 48 h. Bands a–h were cut out, colonized, and sequenced. B) The UPGMA analysis of PCR-DGGE profiles from six fecal samples before and after AO fermentation. (TIF) [file pone.0091106.s001.tif]

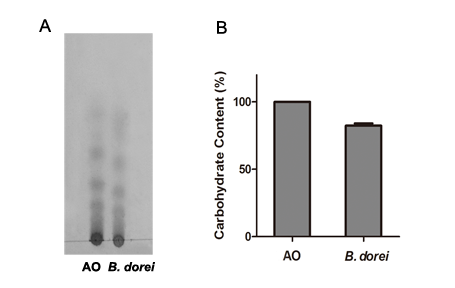

Supplement: Figure S2 — TLC analysis of AO degradation by B. dorei after 192 h of fermentation. A) TLC patterns after AO fermentation by B. dorei. B) The residual values of total carbohydrates after AO fermentation by B. dorei. (TIF) [file pone.0091106.s002.tif]

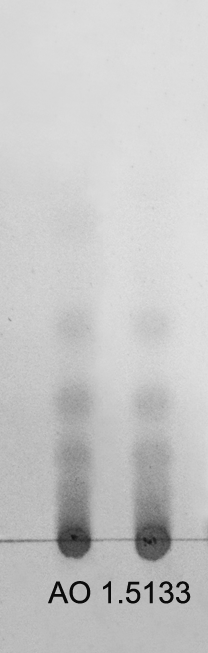

Supplement: Figure S3 — TLC analysis of AO degradation by B. uniformis 1.5133 after 192 h of fermentation. (TIF) [file pone.0091106.s003.tif]

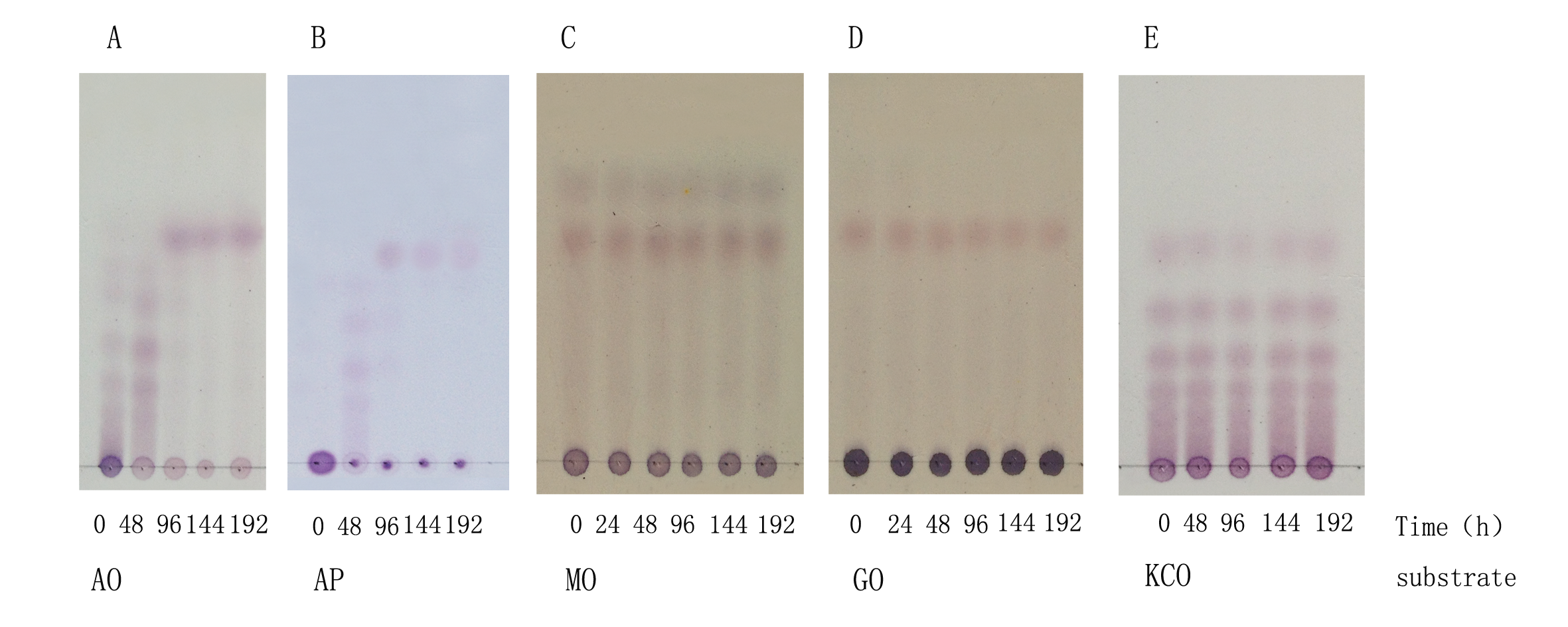

Supplement: Figure S4 — Degradation of AO, AP, KCO, MO, and GO by B. uniformis L8 TLC patterns of AO, AP, KCO, MO and GO fermentation by B. uniformis L8. Samples from AO, AP, and KCO fermentation were collected at 0, 48, 96, 144, and 192 h. MO and GO were collected at 0, 24, 48, 96, 144, and 192 h. (TIF) [file pone.0091106.s004.tif]
