# Supplementary material for: Isolation and Characterization of an Agaro-Oligosaccharide (AO)-Hydrolyzing Bacterium from the Gut Microflora of Chinese Individuals
Source: PLoS One. 2014 Mar 12;9(3):e91106. doi: 10.1371/journal.pone.0091106 (PMC3951304; doi:10.1371/journal.pone.0091106)
Supplement: Table S1 — Primers used for qPCR. (DOCX) [file pone.0091106.s005.docx]

**Table S1. Primers used for qPCR**

| Target group | Primer sequences (5′-3′) | Amplicon size | Annealing temperature | Reference |
| --- | --- | --- | --- | --- |
| *Bacteroides-Prevotella* group | Bac303F: GAAGGTCCCCCACATTG | 418 bp | 56°C | (1) |
|  | Bac708R: CAATCGGAGTTCTTCGTG |  |  |  |
| *Bifidobacterium* genus | Bif164F: GGGTGGTAATGCCGGATG | 440 bp | 59°C | (1) |
|  | Bif601: TAAGCCATGGACTTTCACACC |  |  |  |
| *Clostridium* cluster XⅠVab | CloXIV-F: GAWGAAGTATYTCGGTATGT | 150 bp | 54°C | (2) |
|  | CloXIV-R: CTACGCWCCCTTTACAC |  |  |  |
| *Enterobacteriaceae* | Eco-F: CATTGACGTTACCCGCAGAAGAAGC | 189 bp | 63°C | (1) |
|  | Eco-R: CTCTACGAGACTCAAGCTTGC |  |  |  |
| *Lactobacillus* group | Lac-F: AGCAGTAGGGAATCTTCCA | 344 bp | 58°C | (3) |
|  | Lac-R: ATTYCACCGCTACACATG |  |  |  |
| Total bacteria | Bac1114F: CGG CAA CGA GCG CAA CCC | 145 bp | 66°C | (4) |
|  | Bac1275R: CCA TTG TAG CAC GTG TGT AGC C |  |  |  |

**References:**

1. **Bartosch S, Fite A, Macfarlane G T, and McMurdo M E.** 2004. Characterization of bacterial communities in feces from healthy elderly volunteers and hospitalized elderly patients by using real-time PCR and effects of antibiotic treatment on the fecal microbiota. Appl Environ Microbiol. **70:**3575–81.

2. **Song Y, Liu C, and Finegold S M.** 2004. Real-time PCR quantitation of clostridia in feces of autistic children. Appl Environ Microbiol. **70:**6459–65.

3. **Walter J, Hertel C, Tannock G W, Lis C M, Munro K, and Hammes W P.** 2001. Detection of Lactobacillus, Pediococcus, Leuconostoc, and Weissella species in human feces by using group-specific PCR primers and denaturing gradient gel electrophoresis. Appl Environ Microbiol. **67:**2578–85.

4. **Denman S E, and McSweeney C S.** 2006. Development of a real-time PCR assay for monitoring anaerobic fungal and cellulolytic bacterial populations within the rumen. FEMS Microbiol Ecol. **58:**572–82.
