# Supplementary material for: Isolation and Characterization of an Agaro-Oligosaccharide (AO)-Hydrolyzing Bacterium from the Gut Microflora of Chinese Individuals
Source: PLoS One. 2014 Mar 12;9(3):e91106. doi: 10.1371/journal.pone.0091106 (PMC3951304; doi:10.1371/journal.pone.0091106)
Supplement: Table S2 — Bacterial populations (log cells/ml) and pH values from six fecal slurries after batch fermentation of AO. Samples were taken at 0 and 48 h of AO fermentation. (DOCX) [file pone.0091106.s006.docx]

**Table S2. Bacterial populations (log cells/ml) and PH of batch cultures in the presence of AO at 0 and 48 h**

| Human fecal sample no. | Time (h) | pH | Total bacteria | *Bacteroides-Prevotella* group | *Bifidobacterium* genus | *Clostridium* cluster XIVab | *Enterobacteriaceae* | *Lactobacillus* group |
| --- | --- | --- | --- | --- | --- | --- | --- | --- |
| 1 | 0 | 6.50 | 11.25 | 10.40 | 6.74 | 9.28 | 7.38 | 5.50 |
|  | 48 | 5.71 | 11.32 | 10.70 | 7.25 | 9.50 | 7.49 | 5.35 |
| 2 | 0 | 6.50 | 11.28 | 10.53 | 7.44 | 9.02 | 7.79 | 5.94 |
|  | 48 | 6.49 | 11.07 | 10.47 | 7.82 | 9.27 | 7.77 | 4.95 |
| 3 | 0 | 6.50 | 11.28 | 10.37 | 5.46 | 8.77 | 9.00 | 4.96 |
|  | 48 | 6.33 | 11.40 | 9.94 | 6.03 | 9.34 | 9.66 | 4.85 |
| 4 | 0 | 6.50 | 11.48 | 10.64 | 6.29 | 9.28 | 7.94 | 5.55 |
|  | 48 | 5.80 | 11.19 | 9.76 | 7.08 | 9.11 | 7.11 | 5.36 |
| 5 | 0 | 6.50 | 11.24 | 10.44 | 6.06 | 9.18 | 7.63 | 5.38 |
|  | 48 | 5.39 | 11.58 | 10.93 | 6.57 | 9.36 | 9.20 | 5.49 |
| 6 | 0 | 6.50 | 10.61 | 9.62 | 6.43 | 8.66 | 7.61 | 4.84 |
|  | 48 | 5.77 | 11.51 | 9.88 | 6.68 | 9.35 | 7.07 | 4.95 |
