# Supplementary material for: Isolation and Characterization of an Agaro-Oligosaccharide (AO)-Hydrolyzing Bacterium from the Gut Microflora of Chinese Individuals
Source: PLoS One. 2014 Mar 12;9(3):e91106. doi: 10.1371/journal.pone.0091106 (PMC3951304; doi:10.1371/journal.pone.0091106)
Supplement: Table S3 — Biochemical details of E. coli B2 determined with GN card by VITEK Compact automatic bacteria identification instrument. (DOCX) [file pone.0091106.s007.docx]

**Table S3. Biochemical details of B2 determined with GN card and a VITEK Compact automatic bacteria identification instrument**

| APPA | - | ADO | - | PyrA | - | IARL | - | dCEL | - | BGAL | + |
| --- | --- | --- | --- | --- | --- | --- | --- | --- | --- | --- | --- |
| H2S | - | BNAG | - | AGLTp | - | dGLU | + | GGT | - | OFF | + |
| BGLU | - | dMAL | + | dMAN | + | dMNE | + | BXYL | - | BAlap | - |
| ProA | - | LIP | - | PLE | - | TyrA | - | URE | - | dSOR | + |
| SAC | + | dTAG | - | dTRE | + | CIT | - | MNT | - | 5KG | - |
| ILATK | - | AGLU | - | SUCT | - | NAGA | - | AGAL | + | PHOS | - |
| GlyA | - | ODC | + | LDC | + | IHISa | - | CMT | + | BGUR | + |
| O129R | - | GGAA | - | IMLTa | - | ELLM | + | ILATa | - |  |  |
